# A Combined Plasmonic and Electrochemical Aptasensor Based on Gold Nanopit Arrays for the Detection of Human Serum Albumin

**DOI:** 10.3390/nano13162374

**Published:** 2023-08-19

**Authors:** Ruifeng Zhu, Gabriela Figueroa-Miranda, Lei Zhou, Ziheng Hu, Bohdan Lenyk, Sven Ingebrandt, Andreas Offenhäusser, Dirk Mayer

**Affiliations:** 1 Institute of Biological Information Processing, Bioelectronics (IBI-3), Forschungszentrum Jülich GmbH, 52428 Jülich, Germany; 2 Institute of Materials in Electrical Engineering 1, RWTH Aachen University, 52074 Aachen, Germany; 3 Department of Physics, University of Konstanz, 78457 Konstanz, Germany

**Keywords:** gold nanopit arrays, gold nanohole arrays, surface plasmon polaritons, aptasensor, nanoimprint lithography, nanosphere lithography

## Abstract

Electrochemical and optical platforms are commonly employed in designing biosensors. However, one signal readout can easily lead to inaccuracies due to the effect of nonstandard test procedures, different operators, and experimental environments. We have developed a dual-signal protocol that combined two transducer principles in one aptamer-based biosensor by simultaneously performing electrochemical- and extraordinary optical transmission (EOT)-based plasmonic detection using gold nanopit arrays (AuNpA). Compared with full hole structures, we found that nanopits, that did not fully penetrate the gold film, not only exhibited a better plasmonic bandwidth and refractive index sensitivity both in the finite-difference time-domain simulation and in experiments by shielding the gold/quartz mode but also enlarged the electrochemical active surface area. Therefore, the periodic non-fully penetrating AuNpA were modified with ferrocene-labeled human serum albumin aptamer receptors. The formation of the receptor layer and human serum albumin binding complex induced a conformational change, which resulted in variation in the electron transfer between the electro-active ferrocene units and the AuNpA surface. Simultaneously, the binding event caused a surface plasmon polaritons wavelength shift corresponding to a change in the surface refractive index. Interestingly, although both transducers recorded the same binding process, they led to different limits of detection, dynamic ranges, and sensitivities. The electrochemical transducer showed a dynamic detection range from 1 nM to 600 μM, while the optical transducer covered high concentrations from 100 μM to 600 μM. This study not only provides new insights into the design of plasmonic nanostructures but also potentially opens an exciting avenue for dual-signal disease diagnosis and point-of-care testing applications.

## 1. Introduction

Human serum albumin (HSA) is an abundant protein found in human blood plasma or serum with important physiological functions. HSA constitutes nearly half of the blood serum proteins, ranging from 30 to 50 g/L in serum [1,2,3]. Abnormal HSA levels are associated with coronary heart diseases, multiple myeloma, diabetes, nephropathy, neurometabolic disorders, liver cirrhosis and other diseases [4]. Therefore, accurate and quantitative detection of HSA is highly important for research and applications in biological science, molecular biology, clinical medicine and other fields. The most commonly used determination method for HSA in clinical laboratories is based on bromocresol (BCG) green binding assays. However, this method suffers from the disadvantages of poor selectivity and sensitivity, especially when the sample contains many other proteins [5]. Thus, developing a general and accurate way to determine the HSA content with high selectivity and a broad detection range is of great value.

Recently, nucleic acid aptamers have attracted intense interest since they possess many advantages as recognition elements in biosensing when compared with traditional antibodies, such as being small in size, chemical and thermal stability, having low batch-to-batch variation and low costs. More importantly, aptamers offer remarkable flexibility and convenience in the design of their structures, which has led to novel bioassays that have exhibited high affinity, high sensitivity and selectivity for diverse targets. Up to now, various classes of aptamer-based biosensors (aptasensors) have been designed, including electrochemical, mass-sensitive and optical aptasensors [6,7]. Among them, the electrochemical techniques usually utilize redox groups or molecular beacons to indicate the binding of the biomarker to the aptamer receptor. Although these redox group-based signal strategies have been widely used, false positive or negative detection may occur during recording of the current signal due to several factors, including a low surface density for the aptamer or redox probe, unspecific binding of matrix molecules, biofouling, undesired interactions between the redox probe and receptor molecules and instabilities within the aptamer and blocking molecular receptor layer. Furthermore, degradation of the redox probe can be misinterpreted as an authentic response of the target, generating a false positive signal in particular for electrochemical aptasensors with a “signal-off” mechanism [8]. In response, we established in this work a combination of an electrochemical transducer system with an independent and complementary optical transducer to overcome these signal recording uncertainties and improve the reliability of the detection assay.

Therefore, we employed accompanying measurements of surface plasmon resonance (SPR) signals, which are electromagnetic oscillations of electrons occurring at a metal/dielectric interface and are extremely sensitive to the refractive index of materials immediately adjacent to a thin metal film [9], making them a popular technique in biosensors. Compared with the classical Kretschmann configuration of SPR, the EOT configuration exhibits transmission peaks in the film’s spectrum with significantly higher efficiency than expected according to the standard aperture theory. In this configuration, the light transverses through a subwavelength grating of noble metal nanohole arrays instead of using the bulky prism coupling mechanism [10]. By this means, the complex prism coupling instrumentation is eliminated, which hampers both the high throughput required for multiplexed analysis and the integration into lab-on-a-chip systems [11]. The initial observation of EOT by Ebbesen et al. [12] also provided evidence that this phenomenon is related to the excitation of propagating surface plasmons due to periodically patterned metallic surfaces. Geometrical parameters, such as the symmetry and periodicity of the aperture array, hole diameter and film thickness, play an important role in EOT and enable a fine-tuning of spectral responses [13,14,15]. However, these biosensors are based on full hole geometrical research, and the EOT spectra of full hole geometry have multiple resonance peaks that are spectrally close to each other, which impairs the figure of merit and thus the biosensing applications, requiring reliable and accurate identification of spectral shifts.

**Figure 1:**
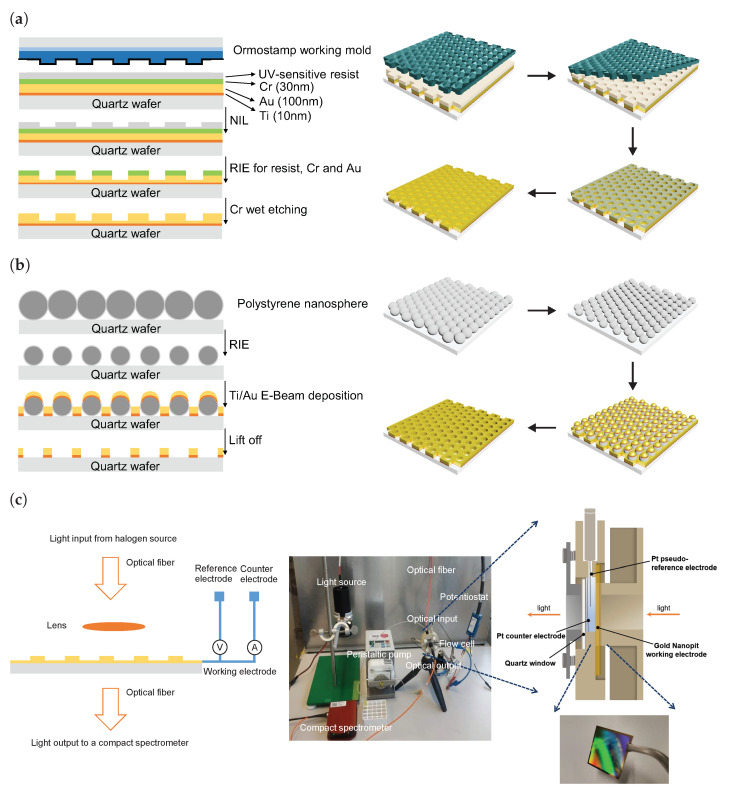
Schematic representation of the fabrication of AuNpA based on nanoimprint lithography (**a**) and full AuNhA based on nanosphere lithography (**b**). Schematic of the simultaneous plasmonic and electrochemical dual signal measurement set-up (**c**).

To overcome the limitations of single transducer sensor concepts [16], our group developed a dual-signal protocol which combines two transducer principles in one aptasensor by simultaneously performing electrochemical and plasmonic detection. Such a configuration was demonstrated by using gold nanohole arrays as a biosensor for malaria biomarker detection [17]. In this work, we use the same dual biosensing transducer principle but utilize AuNpA instead of nanohole arrays. Compared with the well-investigated full hole geometry, the AuNpA is studied here for the first time. We find that AuNpA not only exhibit a sharper plasmonic spectral width and higher refractive index sensitivity in both finite-difference time-domain (FDTD) simulations and experiments by shielding the Au/substrate mode but also enlarge the electrochemically active surface area as nanopatterned electrodes compared with fully gold nanohole arrays (AuNhA) fabricated by nanosphere lithography (Figure 1b) to make sure that the metal layers are fully penetrated. The thin gold films perforated with hexagonal periodic arrays of nanopits were fabricated by nanoimprint lithography (Figure 1a), modified with aptamer receptors and subsequently utilized for the detection of HSA. The AuNpA can be simultaneously utilized as the working electrode for electrochemical measurement and as the metal surface that facilitates the recording of surface plasmon polaritons (SPP) peak shifts of the transmitted light correlated with analyte binding to further improve the diagnosis accuracy and reliability and broaden the range of detection via a dual-signal function. Furthermore, our novel dual transducer aptasensor is highly modular, utilizing a portable photospectrometer, and it can therefore be used for point-of-care testing, different from conventional SPR systems.

## 2. Experimental Section

### 2.1. AuNpA Fabrication Based on Nanoimprint Lithography (NIL)

Master stamp: The master mold decorated by nanopit arrays was made by oxidizing a silicon wafer (4-inch diameter, n-type, <100> orientation and 500–550 μM thickness, Silicon Materials, Pittsburgh, PA, USA) to obtain a top SiO${}_{2}$ layer (dark blue, thickness of 100 nm). A polymethylmethacrylate (PMMA, AR-P 669.04, Allresist GmbH, Strausberg, Germany) resist was spin-coated on the Si/SiO${}_{2}$ wafer and used for electron beam lithography. This involved exposing the resist to an electron beam with an acceleration voltage of 50 kV, followed by a development step. Subsequently, the SiO${}_{2}$ was etched with CH${}_{3}$/SF${}_{6}$ plasma using reactive ion etching (RIE, Oxford PL 100, Oxford Instruments, Abingdon, UK). The final resist removal was performed in the RIE chamber using O${}_{2}$ plasma by cooling the wafer to −140 °C.

The master mold surface was further treated by growing an anti-adhesive layer on top with a vapor-phase silanization process. For this purpose, the mold was cleaned and activated using an O${}_{2}$ plasma treatment (Pico low-pressure plasma, Diener Electronic GmbH, Ebhausen, Germany) for 3 min at 80 W of power and 0.7 mbar of pressure to facilitate the covalent binding of the FOTCS (trichloro (1H,1H,2H,2H-perfluorooctyl) silane) molecules via silanol groups to the SiO${}_{2}$ surface. The wafer was therefore transferred to an argon atmosphere glove box (99.99% argon atmosphere, MB200B, MBRAUN, Garching, Germany) equipped with a desiccator for the silanization step. Specifically, 200 μL of FOTCS was transferred to the gas phase at a pressure of 45 mbar and deposited on the surface for 1.5 h to form a homogeneous self-assembled monolayer (SAM). After surface modification, the wafer was rinsed in an acetone, isopropanol and water cascade before replication.

Working stamp: The fabrication process of the NIL working stamp is illustrated in Figure S1. First, a transparent 4-inch quartz wafer was treated in oxygen plasma using a Gigabatch system (TePla Gigabatch 360, PVA MPS GmbH, Wettenberg, Germany) for 5 min (O${}_{2}$, 600 sccm, 600 W) in order to remove any organic contamination from the surface. Then, an adhesive promoter, Ormosprime 08 (Micro resist technology GmbH, Berlin, Germany), was coated on the quartz wafer to increase the adhesion of the Ormostamp polymer. Ormosprime was spin-coated at 4000 rpm for 60 s with an acceleration of 1000 rpm/s and hardbaked for 5 min at 150 °C (film thickness of 130±15 nm according to the datasheet in Table S1).

The next step was to dispense 80 μL of Ormostamp resist (Micro resist technology GmbH, Berlin, Germany) on the 4-inch master mold modified with the FOTCS anti-adhesive layer. Then, the quartz wafer was placed on top of the Ormostamp droplet (avoiding small bubbles as much as possible). As soon as the wafer was in contact with the droplet, the Ormostamp started to spread until the gap between the master stamp surface and the backplane was completely filled. The low viscosity of the Ormostamp resist facilitated the efficient filling of the master stamp nanopit structure and minimized the propensity to form air bubble defects. The transferred negative replica from the master stamp was solidified by a UV flood exposure (MA-6 Mask Aligner under 1000 mJ/cm${}^{2}$ for 2 min, Süss Microtech AG, Garching, Germany), and a final thermal post-baking treatment was implemented at 130 °C for 30 min. A similar treatment with the FOTCS anti-sticking agent was carried on for the Ormostamp surface in a glove box.

Nanoimprinting process: NIL is widely regarded as a mature and highly effective technique for achieving nanometer-scale features on large wafers. It offers several advantages, including higher throughput, cost-effectiveness, compatibility with large areas and excellent uniformity [18]. The NIL process is sketched in Figure 1a. First, a tri-metallic layer (10 nm Ti, 100 nm Au and 30 nm Cr as a hard mask layer) was evaporated (PLS 570, Pfeiffer Vacuum, Aßlar, Germany) onto the quartz wafer. The mr-NIL200 (Micro resist technology GmbH, Berlin, Germany) is a photo-curable NIL resist specifically suited for applying hard and non-permeable stamp materials. Before coating, all substrates were treated with a baking step at 110 °C for 3 min to evaporate the organic solvent. Then, the mr-NIL200 resist was spin-coated on the quartz/Ti/Au/Cr surface at 3000 rpm for 30 s with an acceleration of 1000 rpm/s followed by a soft bake for 3 min at 60 °C. The resist’s thickness was 200 nm, as determined by an SE800 ellipsometer (Sentech GmbH, Berlin, Germany). Nanoimprint lithography (NX-2000, Nanonex, Monmouth Junction, NJ, USA) was used to produce the patterned quartz/Ti/Cr/Au/mr-NIL200 wafer. The quartz/Ti/Cr/Au/mrNIL200 wafer and the working stamp wafer were placed together between two silicone foils and compressed by the air cushion principle. A pre-imprint lasting 1 min at 100 psi was necessary for stabilization and homogeneity of the process. The main imprint was performed for 5 min at 200 psi, followed by UV irradiation (emitted light wavelength of 365 nm) for 1 min. All imprint processes were conducted at room temperature. The wafers were detached softly with a razor blade. The final patterned quartz/Ti/Cr/Au/mr-NIL200 wafer was prepared for further etching steps.

Etching process: After the UV-NIL process, three steps of RIE were performed, including O${}_{2}$ plasma etching of the mr-NIL200 residual layer first, Cl${}_{2}$/O${}_{2}$ etching of the Cr hard mask second and finally Cl${}_{2}$/Ar etching of the Au. Considering the selectivity of mr-NIL200 over the Cr layer and Cr over the Au layer, different etching recipes for different layers were chosen. Directly etching the Au layer using only the mr-NIL200 resulted in shallow etching depths. Cr is one of the most popular hard etching mask materials due to its high resistance to plasma etching. Plasma etching of Cr has been well established, utilizing Cl-containing gases (e.g., Cl${}_{2}$ and CCl${}_{4}$) and oxygen [19]. The etching characteristics of Au with Cl${}_{2}$/Ar-based chemistry take place through a combination of ion-assisted chemical reactions and physical sputtering. The etching temperature needs to be carefully controlled to maintain the volatility of AuCl${}_{3}$ while avoiding its thermal decomposition to non-volatile AuCl [20]. The parameters for the dry etching steps were determined by a Dektak profilometer (DektakXT, Bruker, Billerica, MA, USA) and are summarized in Table S2.

Once the dry etching was complete, the Cr residual layer was removed using a Cr etchant, resulting in a thin Au layer perforated with nanopit arrays. The prepared AuNpA were rinsed in an acetone, isopropanol, and water cascade and then treated with oxygen plasma, ethanol and Milli-Q water before they were finally dried with N${}_{2}$ for further use. The etching process was similar to the previously reported work by Robinson et al. [21]. It is noteworthy that it is challenging to etch through the entire gold layer down to the quartz wafer to generate a full hole structure without over-etching. In contrast, nanopit arrays can be easily achieved and possess advantages over nanoholes regarding plasmon excitations by eliminating the Au/substrate mode.

### 2.2. AuNhA Fabrication Based on Nanosphere Lithography (NSL)

To obtain the AuNhA structure for comparison, nanosphere lithography was employed on 4-inch quartz wafers (thickness of 100.00 × 0.525 mm, Wafer Universe, Elsoff, Germany) in a manner similar to what our group previously reported [22,23,24] by utilizing the PE funnel-assisted interfacial assembly approach as shown in Figure 1b. Briefly, the quartz wafer as a substrate was first cleaned in oxygen plasma at 0.7 mbar with 200 W for 5 min to hydrophilize the surface for the successive nanosphere lithography. Then, a beaker was filled with Milli-Q water, and the wafer was placed onto the sample stage within a PE funnel. A small amount of surfactant (20 μL TX-100, 10 mM) was added directly to the surface of the Milli-Q water in order to improve particle packing. After that, 0.8 mL of 2.5% polystyrene nanosphere dispersion (Bangs Laboratories, Fishers, IN, USA) in a mixture of Milli-Q water and absolute ethanol (*v*/*v*, 1:1) was slowly dropped onto a glass slide such that it flowed to the surface of the water. Subsequently, the sample was dried by pumping out the water through a tube leading to a homogeneous, ordered and closely packed hexagonal particle film. Later, RIE was employed to reduce the particle sizes such that a non-close-packed particle array was formed. The etching gas had a composition of O${}_{2}$ and CHF${}_{3}$ with a ratio of 40:10 sccm. The etching process was conducted at 0.026 mbar, 0 °C and a radio frequency (RF) power of 30 W for 6 min.

In the subsequent metallization step, two metal layers, 10 nm Ti and 100 nm Au, were deposited onto the wafer using electron beam evaporation with deposition rates of 0.1 and 0.5 nm/s, respectively. The polystyrene particles were removed using adhesive cello tape, leading to the formation of full AuNhA on the quartz substrate. Then, the AuNhA were successively sonicated with acetone and isopropanol for 5 min, followed by rinsing with Milli-Q water and drying in an N${}_{2}$ stream. Finally, the AuNhA was treated with oxygen plasma (O${}_{2}$, 0.5 mbar, 50% power with a duration of 3 min), washed with ethanol under sonication for another 5 min to reduce the formed gold oxides, rinsed with Milli-Q water and dried with N${}_{2}$ for further use.

### 2.3. Plasmonic and Electrochemical Dual-Signal Measurement Set-up and Data Acquisition

In the optical experimental set-up, as shown in Figure 1c, we used an unpolarized halogen lamp, since the nanoholes were symmetric and the transmittance at normal incidence was independent of the polarization, which eliminated the need for a linear polarizer. The light transmitted from the AuNpA was coupled to a compact photo-spectrometer (Thorlabs CCS200, Newton, NJ, USA). The EOT spectra were determined by finding the ratio between the spectra of the transmitted light from the Tris buffer solution with modified AuNpA and the blank Tris buffer solution. In the plasmonic characterization, the integration time was 1.8 ms and was recorded with 200 spectrum frames. A custom Python script using the sklearn package was applied to fit a third-order polynomial function around the average fitted SPP peak and track the peak wavelength value. Simultaneously, electrochemical experiments were performed on a three-electrode spectro-electrochemical flow cell with a reduced optical path (Redoxme AB, Norrköping, Sweden) at room temperature. The flow cell consisted of a polyether ether ketone flow chamber equipped with a magnetic mount sample holder for an electrode with a size of 25 × 25 mm${}^{2}$. The total internal volume of the chamber was 0.7 mL, which allowed shortening the optical path to 4 mm. The AuNpA samples were first cleaned using acetone and isopropanol through sonication for 5 min and then rinsed with Milli-Q water and dried with N${}_{2}$. Next, an oxygen plasma treatment was applied (O${}_{2}$ pressure of 0.5 mbar at 50% power for 5 min), followed by immersion in ethanol to reduce any formed gold oxides. Finally, the AuNpA were dried with N${}_{2}$ to complete the cleaning process and mounted to the cell as the working electrode. A Pt wire immersed in the sample solution was used as a pseudo-reference electrode (50 mm long) to minimize the distance to the working electrode, and a Pt electrode was employed as counter electrode in the form of a Pt wire (90 mm long). The positions of three electrodes are illustrated on the right side of Figure 1c. Alternating current voltammetry (ACV) measurements were performed using an Autolab PGSTAT302 (Metrohm, Herisau, Switzerland) with NOVA software (2.1v) in a Tris buffer (10 mM Tris, 150 mM NaCl, 5 mM KCl, pH 7.4). The ACV scans had a rate between −0.1 and 0.7 V with potential steps of 0.01 V, a modulation amplitude of 0.04 V, modulation time of 0.4 s, frequency of 20 Hz and interval time of 0.8 s.

### 2.4. Preparation of the Aptasensor

In this work, the HSA aptamer (5${}^{\prime }$-Ferrocene-GTC TCA GCT ACC TTA CCG TAT GTG GCC CAA AGC GTC TGG ATG GCT ATG AA-(CH${}_{2}$)${}_{6}$-SH-SH-3${}^{\prime }$) [25] was HPLC-purified and obtained from Sangon Biotech (Shanghai, China). The concentrations of the aptamers were determined with a DS-11 series spectrophotometer (DeNovix, Wilmington, DE, USA). All the solutions were prepared with Milli-Q water purified through a Milli-Q ultrapure water system (18.25 MΩ cm, Gradient A10, Merck Millipore, Burlington, Burlington, NJ, USA). The 20 nM aptamer solution [26] was first activated in a high-salt Tris buffer (10 mM Tris, 1.5 M NaCl, 1 mM MgCl${}_{2}$, pH 7.4) with 10 mM tris(2-carboxyethyl)phosphine (TCEP) solution (at a molar ratio of 1:1000) for 1 h to cleave the disulfide bonds. The nanostructured samples were incubated with the above solution at room temperature to immobilize the molecules on the surface through Au-S bonds by flushing the molecules over the surface with a peristaltic pump for 16 h. Then, the samples were thoroughly rinsed with the Tris buffer to remove the non-bonded aptamer. Subsequently, the as-prepared samples were incubated with 0.5 mM 6-mercapto-1-hexanol (6-MCH) for 1 h to obtain a compact, self-assembled receptor layer by blocking the unmodified electrode sites and suppressing unspecific binding. Lastly, a thorough rinsing step with the Tris buffer was performed to remove blocking molecules that were not covalently bound. For the HSA assay, the aptasensor was incubated in various concentrations of the analyte molecules for 30 min. After incubation, the nanostructured Au electrodes were rinsed with the Tris buffer to remove HSA molecules that were not specifically adsorbed.

### 2.5. Atomic Force Microscopy Measurements

Atomic force microscopy (AFM) imaging was performed using a Bruker Nanoscope V Multimode AFM set-up (Bruker, Billerica, MA, USA) to characterize the surface topography with a high resolution. The images were acquired in tapping mode with tesp-v2 cantilevers (tip radius of 7 nm, length of 123 μm and width of 40 μm; Bruker, Billerica, MA, USA).

### 2.6. Finite-Difference Time-Domain Simulations

Numerical finite-difference time-domain (FDTD) simulations (2021 R1.4, Lumerical Solutions) were utilized to optimize the transmission spectra of different geometrical parameters and localize the distribution of the electric fields on the gold nanostructures. The models for the quartz, Ti and Au layers were taken from the default database. Perfectly matched layer boundary conditions were used along the propagating direction (z direction). To reduce the simulation volume and time, anti-symmetric and symmetric boundary conditions were applied along the x and y directions, respectively. The spatial steps of the discrete mesh in the simulation were set to Δx = Δy = 5 nm and Δz = 2.5 nm.

## 3. Results and Discussion

### 3.1. Periodicity Optimization by FDTD Simulation

The occurrence of EOT, in which the transmission spectrum of a film shows peaks that surpass the expected transmission efficiency according to the standard aperture theory, arises from a complex mechanism of surface plasmon excitation, depending on the geometric structure of the sample surface [27]. Surface plasmon polaritons (SPP) can be excited when the oscillations of surface charges at the metal interface once the wave vector of the incident photon matches the grating on the metal according to Equation (1) [12,28,29]:

(1) $${\vec{k}}_{sp}={\vec{k}}_{x}\pm i\vec{G_{x}}\pm j\vec{G_{y}}$$

According to the dispersion relation for the surface plasmon, ${\vec{k}}_{sp}=2\pi /\lambda \sqrt{\varepsilon_{m}\varepsilon_{d}/\left(\varepsilon_{m}+\varepsilon_{d}\right)}$ is the surface plasmon wave vector, where $\varepsilon_{m}$ and $\varepsilon_{d}$ are the dielectric constants of the metal and dielectric layer, respectively, ${\vec{k}}_{x}=\left(2\pi /\lambda \right)\sin\theta$ is the component of the incident photon’s wave vector in the plane of the grating, θ is the angle of incidence with respect to the interface normal, ${\vec{G}}_{x},{\vec{G}}_{y}=4\pi /\sqrt{3}P$ are the reciprocal lattice vectors of the hexagonal array of nanoholes and *i* and *j* are integers representing the scattering orders of the array.

Nanohole arrays are nanostructures fabricated on metallic materials featuring a high aspect ratio and large electric field intensities in the subwavelength regime based on EOT. The utilization of metallic surfaces with EOT enables the excitation of surface plasmons using incoherent sources of light. The spectral analysis of the transmission peaks, resulting from changes in the local refractive index, can be monitored through a portable spectrometer utilizing various plasmonic modes [30]. In the case of SPP sensors employing a periodic hexagonal array of nanoholes under normal incidence, Equation (1) can be reduced, and the resonance wavelength can be determined using Equation (2) [13,31]:

(2) $$\lambda_{\mathrm{SPP}}=\frac{P}{\sqrt{\frac{4}{3}\left(i^{2}+ij+j^{2}\right)}}\sqrt{\frac{\varepsilon_{m}n^{2}}{\varepsilon_{m}+n^{2}}}$$

where *n* is the refractive index of the dielectric layer. The refractive index sensitivity can be obtained by using the first-order derivatives of Equation (2) over n for an SPP sensor as shown in Equation (3):

(3) $$S=\frac{d\lambda_{\mathrm{SPP}}}{dn}=\frac{P}{\sqrt{\frac{4}{3}\left(i^{2}+ij+j^{2}\right)}}\sqrt{{\left(\frac{\varepsilon_{m}}{\varepsilon_{m}+n^{2}}\right)}^{3}}$$

This equation indicates that the refractive index sensitivity of SPP modes is mainly determined by the periodicity and the scattering orders, although it is also known that the aperture size and the type of metallic nanostructure affect the properties of the EOT [32]. For simplicity, we first simulated a hexagonal arrangement of AuNhA with 10 nm Ti as the adhesion layer, 100 nm Au and a fixed periodicity over the diameter (P/D = 1.5) from 400 nm to 800 nm. The periodicity (lattice constant), hole diameter and total hole depth were labeled as P, D and HD (HD = 110 means it was a full hole structure on both the Ti and Au layers), respectively. The simulation results are shown in Figure S2, where we focused on the (1,0) Au/medium mode, which was more sensitive than the (1,0) Au/substrate mode, because it possessed highly accessible and large local electromagnetic fields, resulting in a stronger spatial overlap between the optical fields and biomolecules [33,34]. When the periodicity was 400 nm, there was only one broad (1,0) Au/medium and Au/substrate hybrid peak which shifted with an increasing refractive index. At a periodicity of 500 nm, the (1,0) Au/medium mode was difficult to recognize because it was covered by the broad (1,0) Au/substrate mode in the medium, while for periodicities of 600 nm, 700 nm and 800 nm, the (1,0) Au/medium mode was easy to identify. We found that the larger the periodicity, the higher the sensitivity, as summarized in Table 1. However, considering the detection window of our optical spectrometer (CCS200 compact spectrometer with an extended range of 200–1000 nm) and the real measurement in an aqueous medium with a refractive index of around 1.33, a periodicity of 600 nm represented the best choice, which not only facilitated a high sensitivity and transmittance but also had the highest figure of merit (FOM) value (11.85 RIU${}^{-1}$). The latter is defined as the ratio of the sensitivity (S) over the full width at half maximum (FWHM) at the resonant peak (FOM = S/FWHM [35]).

### 3.2. AuNhA Characterization

**Figure 2:**
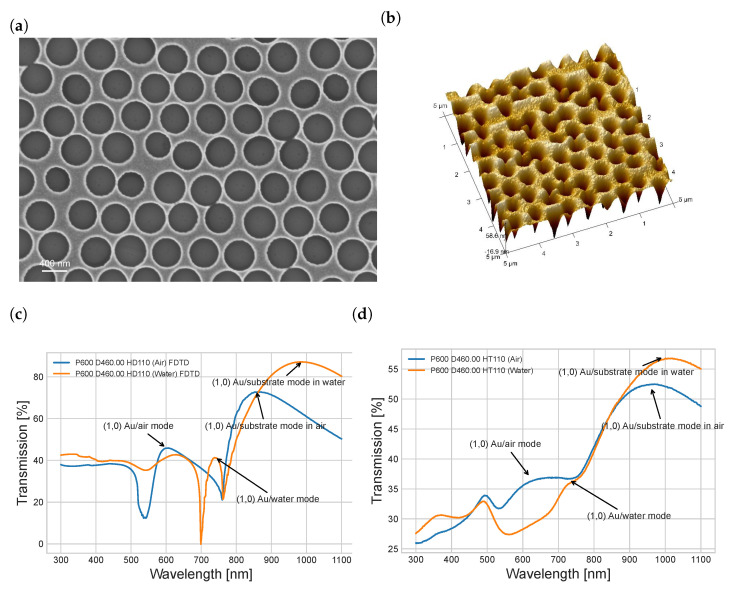
SEM (**a**) and AFM (**b**) images of the full AuNhA (periodicity of 600 nm) fabricated by NSL, FDTD simulation spectrum (**c**) and measured transmission spectrum (**d**) in air and water medium with P (600 nm), D (460 nm) and HD (110 nm).

AuNhA with a periodicity of 600 nm were fabricated using the NSL technique in order to make sure that the Ti and Au layers were fully penetrated by the hole. Figure 2a,b showed representative AFM and SEM images of the AuNhA after the lift-off and cleaning procedure. As expected, the samples consisted of regular hexagonal nanohole patterns with an average periodicity of 601.3 ± 65.3 nm and a diameter of 459.5 ± 28.7 nm. The hole thickness was extracted from the AFM measurements and determined to be 110.7 ± 5.6 nm. This diameter was larger than what we simulated before. Therefore, we performed additional FDTD simulations using AuNhA with a diameter of 460 nm surrounded by air and water as dielectric layers (Figure 2c). Compared with the real measured transmission, as shown in Figure 2d, the (1,0) Au/medium mode was not easy to recognize, especially in water. A similar discrepancy between simulated and measured transmission was found in the recently published work of Zhang et al. [36], where the Au/water (1,0) SPP mode was not prominent using AuNhA with a periodicity of 520 nm and a diameter of 350 nm, in contrast to their simulation spectra. One possible reason for this could be that the peak of the (1,0) Au/substrate mode was too broad to shield parts of the adjacent (1,0) Au/medium mode peak. To solve this problem for better EOT transmission sensing, we suggest reducing the (1,0) Au/substrate mode by eliminating the periodic nanostructure array at the Au/substrate interface by establishing a continuous closed gold film at the interface between the Au and quartz. We manufactured such samples without fully penetrating nanopits in the gold film by simply stopping the Au etching process before reaching the bottom of the Au layer. The resulting AuNpA array facilitates excitation of the (1,0) Au/medium mode, which is required for the sensing performance, but avoids the interfering (1,0) Au/substrate mode.

### 3.3. Different Hole Depths (HDs) Simulated with FDTD

**Figure 3:**
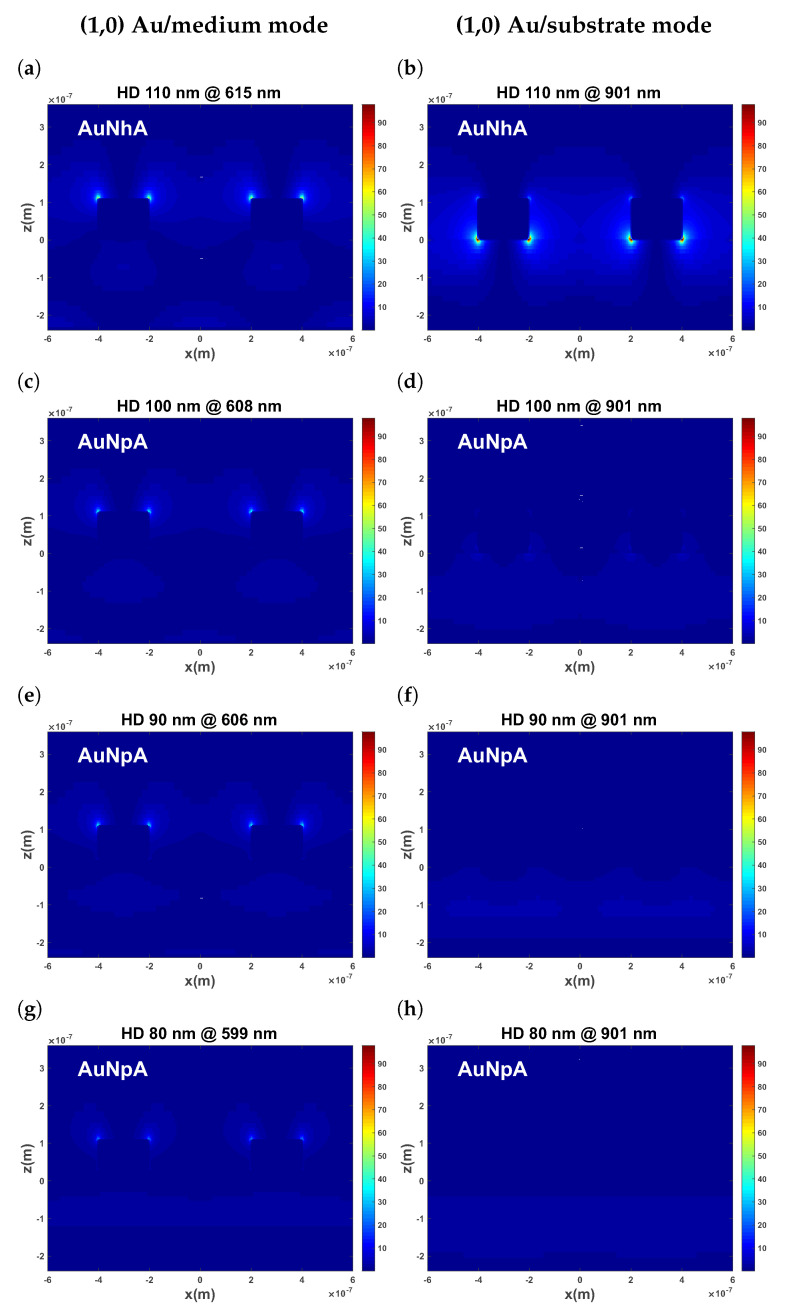
Cross-sectional electric field distribution of (1,0) Au/medium mode (**a**,**c**,**e**,**g**) and (1,0) Au/substrate mode (**b**,**d**,**f**,**h**) when hole depth varies from 110 nm to 80 nm.

In order to verify our assumption, we systematically investigated the influence of the hole depth (HD) changing from 80 nm to 110 nm on the spectrum with FDTD spectrum simulations (Figure 3). For the fully penetrating hole structures (HD = 110 nm), the highest electric field intensity was observed at the top of the gold ring hole for the (1,0) Au/medium mode at 615 nm. Importantly, the confined local electromagnetic fields at the top gold surface extended from the electrode’s vicinity into the medium, which contributed the most to the local refractive index changes during the analyte sensing process. In contrast, for the (1,0) Au/substrate mode at 901 nm, the nearfields were mostly concentrated at the Au and quartz interface, meaning they played a minor role in the biomolecular recognition reactions and the surface binding events.

**Figure 4:**
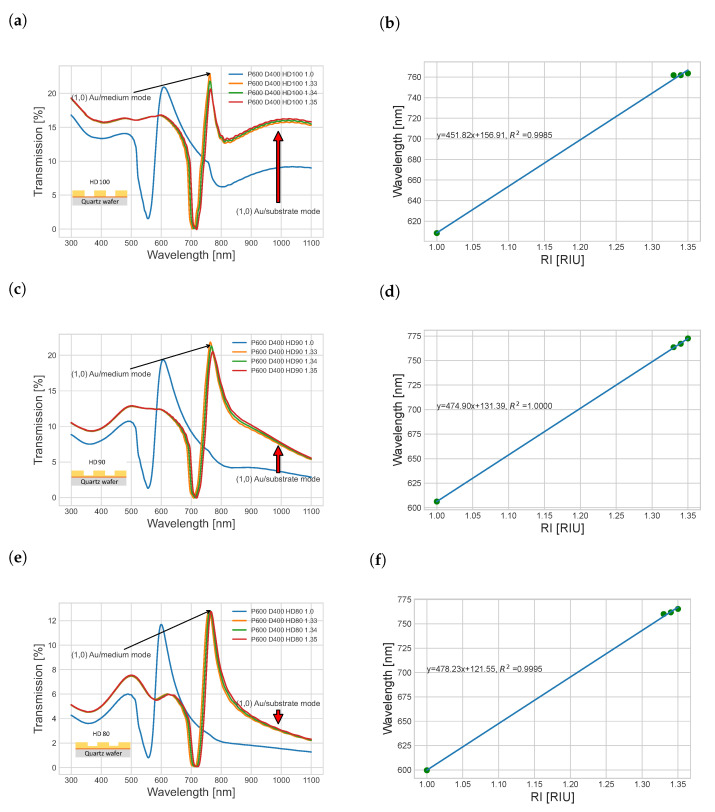
FDTD simulation spectrum with different hole depths from 100 nm, 90 nm and 80 nm (**a**,**c**,**e**, respectively) and refractive index sensitivities of the (1,0) Au/medium mode (**b**,**d**,**f**, respectively).

As the HD decreased, the Au/substrate mode’s intensity also decreased. When the HD reached 80 nm, there was still a 20 nm continuous Au layer on top of the 10 nm Ti. The electric field of the Au/substrate mode can barely be seen, indicating that this mode was effectively suppressed. At the same time, the Au/medium mode intensity remained nearly constant, as expected. We also simulated the spectrum for dielectric media of different refractive index (air (1.0) and water (1.33)) as shown in Figure 4 and Figure S2e,f. The (1,0) Au/medium mode became more distinct, especially compared with the spectrum of the fully penetrating hole configuration. For an HD of 100 nm, there were still faint Au/substrate mode peaks around 1000 nm. Although the transmittance decreased to some extent, the refractive index sensitivity was more important for the wavelength shift sensor, which increased when the HD decreased from 100 nm to 80 nm, as shown in Table 2. Overall, the change in the sensitivity was relatively small for the HD, while the FWHM varied considerably. Consequently, a thickness of 90 nm or 80 nm seems to be a reasonable compromise between the transmittance and FWHM, because a low transmittance causes high noise levels and impairs peak fitting.

### 3.4. AuNpA Characterization

**Figure 5:**
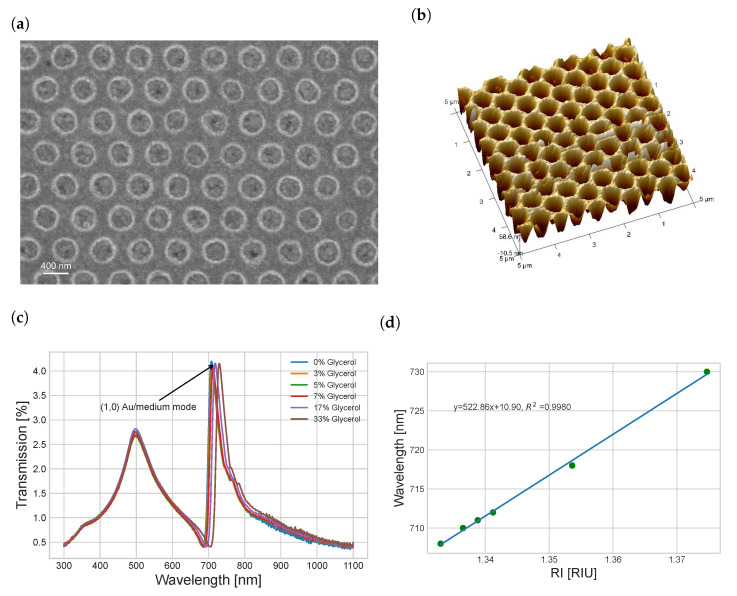
SEM (**a**) and AFM (**b**) images of the AuNpA fabricated by NIL, experimental refractive index sensing (**c**) over a wide concentration range of glycerol aqueous solutions (mass percentage) and peak wavelength regression fitting as a function over the refractive index (**d**).

The surface morphology of the NIL-fabricated AuNpA was characterized by SEM and AFM analysis. The gold film was not fully etched through, and a residual continuous layer remained according to FIB cross-section images (Figure S3). Also, curved side walls were observed, resulting from the isotropic dry etching process. The average periodicity was 600 ± 20 nm, and the diameter was 397 ± 18 nm, as shown in Figure 5a. The larger hole diameter occurred because of the isotropic chemical etching compared with the diameter of the mold SEM image in Figure S4. Before the wet etching, the hole thickness was measured to be 98 ± 2 nm, as shown in Figure S5, while the hole thickness was measured to be 87.9 ± 8.7 nm in the AFM image (Figure 5b) after wet etching.

Figure 5c,d showed the transmission spectra measured after immersing the AuNpA in a sequence of mixed water and glycerol solutions with different mixing ratios and an increasing refractive index from 1.333 (pure water) to 1.375 (33% glycerol). We observed a significant red shift of the peak corresponding to the (1,0) Au/medium mode with an increasing refractive index in the surrounding solution. The (1,0) Au/medium mode sensitivity reached a value of 522.86 nm/RIU. The higher bulk sensitivity may also be explained by the presence of cavity resonance effects from the nanopit structures, while this was not present to such a degree in the apertures [37] As expected, the (1,0) Au/substrate mode was almost completely shielded, which was consistent with our simulation result and ideal for the aspired optical sensing. The nanopit array is also beneficial for electrochemical sensing since it increases the electrochemical surface area (ESA) if we take the ideal side wall area (Figure S6a) of the nanopits into account. The surface area increased by 32% or 36% compared with a compact gold film, assuming a pit depth of 80 nm or 90 nm, respectively, with a lattice constant of 600 nm and a hole diameter of 400 nm according to our calculations. Increasing the effective surface area by converting it from a 2D planar gold film to 3D AuNpA raised the number of receptor binding sites and thereby enhanced the probability of target capturing [38]. However, the experimental data (Figure S6b) indicated that the determined ESA of the AuNpA was only 1.037 times higher than that of the compact Au film. It is plausible that the discrepancy between the calculated and experimental results can be attributed to deviations from the ideal side wall structure, as evidenced by the cross-sectional FIB image of the AuNpA shown in Figure S3. Given these observations, it is essential to acknowledge that for most practical cases, the incremental increase in ESA achieved with AuNpA may be deemed inconsequential. While the nanoscale architecture of the AuNpA provides advantages in certain applications, the relatively small ESA enhancement should be taken into account when assessing the performance and potential benefits of this structure in electrochemical sensing applications.

### 3.5. Performance of Aptasensors for HSA

The performance of the aptasensor critically depends on the proper assembly of the receptor layer on the nanostructured electrode. EIS measurements were conducted to monitor the biosensor fabrication and the subsequent self-assembly processes, utilizing 5 mM [Fe(CN)${}_{6}$]${}^{3-/4-}$ as a redox probe (Figure S7). The obtained impedance spectra were analyzed by fitting them based on a Randles equivalent circuit comprising the serial electrolyte resistance (*R*s), the charge transfer resistance (*R*ct) and a constant phase element (CPE), which improved the fit of the impedance measurements by taking the inhomogeneities of the electrode’s surface into account. Furthermore, a Warburg impedance was used, accounting for the diffusion of the redox probes to the electrode’s surface. The charge transfer resistance of the bare AuNpA electrode was small, having a value of 43 Ω, which increased to 818 Ω and 1290 Ω after aptamer immobilization and further 6-MCH blocking, respectively. The impedance increased because the self-assembled monolayer of the aptamer and 6-MCH blocked the charge transfer. Finally, an increase in *R*ct to 1550 Ω was observed after the addition of 1 μM HSA, indicating binding between the aptamer and target protein due to steric hindrances of the charge transfer. Furthermore, cyclic voltammetry (CV) was employed to investigate the redox behavior of 5 mM [Fe(CN)${}_{6}$]${}^{3-/4-}$ in a Tris buffer solution by using the bare AuNpA as the working electrode under 100 continuous cycles (Figure S8). Remarkably, the obtained results demonstrate that the current response exhibited minimal variation throughout the cyclic voltammograms, indicating high stability for the electrode–electrolyte interface. This finding suggests that the bare AuNpA electrode effectively facilitated the electron transfer reactions and maintained a consistent current response. Optimization of the aptamer incubation concentration, incubation time, blocking molecule immobilization, testing of the selectivity and testing in biological samples was reported in our previous publication [26].

**Figure 6:**
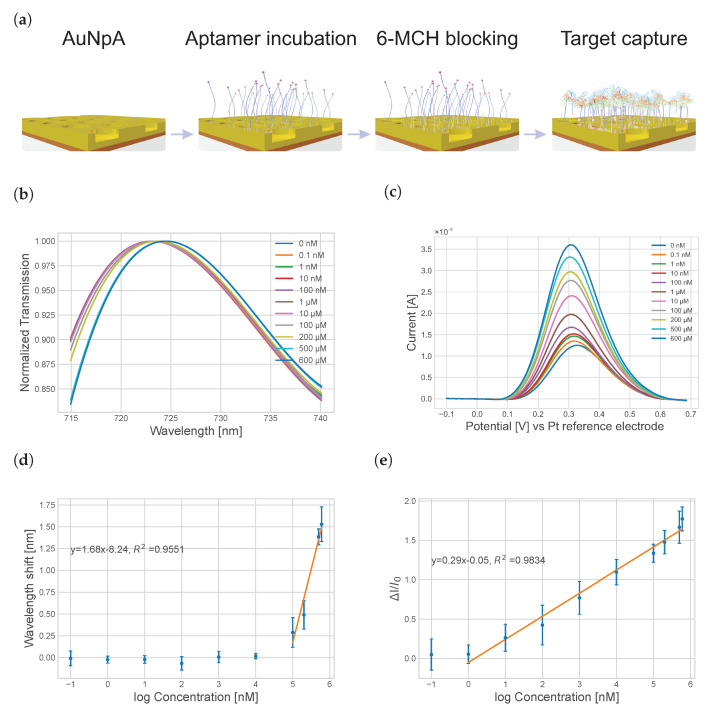
Schematic illustration of the working principle for HSA detection based on non-fully penetrating AuNpA (**a**). The binding of the HSA analyte caused a change in the aptamer conformation, through which the ferrocene redox probe approached the electrode surface and enhanced the charge transfer, fitting the normalized transmission spectrum (**b**) and ACV current responses (**c**) of our aptasensor with different HSA concentrations from 0 to 600 μM. The calibration curve of the sensor was found by plotting the optical shift (**d**) and current change (**e**) versus the logarithm of the HSA concentration, using 0 nM as a reference.

Similarly, the individual steps of the biosensor fabrication (Figure 6a) can be monitored by EOT experiments. Those measurements are convenient and easy to perform by illuminating the AuNpA sample with a probe fiber and collecting the transmitted light from the opposite side. Immobilization of the aptamer receptor and blocking with 6-MCH on the gold surface caused a red shift in the SPP peak (Figure 6b). When the analyte was added to the as-prepared aptasensor, the SPP peak further shifted to a higher wavelength as the HSA concentration increased, indicating the formation of aptamer-target protein complexes on the nanopit arrays. It is noteworthy that a relatively high concentration of analyte molecules is required to induce a significant peak shift. A semi-logarithmic relation between the peak position and concentration was observed for a concentration window between 100 and 600 μM (Figure 6d). Nevertheless, concentrations exceeding 600 μM were not assessed, owing to the fact that the target substance reached its solubility threshold in the solvent utilized for the measurements. The detection limit was 95.22 μM, which was calculated using the formula $S_{\mathrm{dl}}=S_{reag}+3\sigma_{reag}$, with $S_{reag}$ being the averaged signal of the analyte-free solution and $\sigma_{reag}$ representing the standard deviation [39].

For the simultaneous electrochemical characterization of the analyte binding, ACV was used, which facilitated the separation of signals coming from the charging of the electrochemical double layer and the Faradaic currents associated with the redox probes. The signal recording conditions were chosen such that the charging currents were suppressed. When HSA was added, the formation of the aptamer/HSA binding complex induced a conformational change, reducing the distance between the electro-active ferrocene units and the AuNpA surface, consequently causing an increase in electron transfer. A clear increase in the redox peak was observed with increasing HSA concentrations (Figure 6c). The signal gains of the ACV measurements were calculated from the ratio between the current signal drop $\left(\Delta I=I-I_{0}\right)$ and background signal ($I_{0}$) according to the following formula: signal gain (%) = $100\times \left(I=I-I_{0}\right)/I_{0}$. The sensor showed a semi-logarithmic concentration dependence in a wide concentration range from 0.1 nM to 600 μM for the recorded ACV signals (Figure 6e), with a detection limit of 0.08 nM. The characteristics of some previous studies for the quantitative analysis of HSA are presented in Table S3. In comparison with those detection concepts, our dual-signal-based AuNpA exhibited wider dynamic detection ranges than other sensors. Interestingly, the simultaneously recorded signals from both transducers showed different sensitivities. While the electrochemical transducer facilitated a determination of the concentration in a wide concentration range (approximately four orders of magnitude) with a low sensitivity of 0.3/log *C*, the optical transducer showed a higher sensitivity of 1.5/log *C* but exclusively for high concentrations. It is noteworthy that the physiological concentration of HSA in the blood is in the range of hundreds of µM for healthy people [3], which can be covered by our dual-signal aptasensor.

To further evaluate the applicability of our aptamer sensor in more complex matrixes, recovery experiments were performed by the standard addition method in 100-fold human serum mixed with a Tris buffer (pH: 7.4). Both the plasmonic and ACV signal responses are summarized in Table S4, and the reached recoveries for the 100 μM HAS concentrations were 95.30 % and 83.07 %, respectively. The lowers recoveries of 86.19 % and 79.7 % at 200 μM HAS may be attributed to the fact that 6-MCH as a blocking agent is not fully efficient in suppressing electrode fouling, especially in complex blood samples [40]. Despite the slightly lower recoveries, the overall results still indicate good accuracy and reproducibility of the prepared aptasensor.

The reusability of biosensors is an important aspect to consider. In light of this, it is pertinent to mention a specific work [41] that elucidates the effects of various cleaning methods on nanohole arrays. The study indicates that aggressive cleaning methods such as sulfochromic acid, piranha and dry oxygen plasma can lead to irreversible alterations in the nanohole arrays, resulting in irreparable changes to their optical properties and sensitivity and the shape and position of the SPR peak. Conversely, the use of Radio Corporation of America Clean 1 (RCA1 ) etching followed by a diluted HNO${}_{3}$(aq) acid soak has been shown to be a more favorable approach, as it effectively removes surface-bound alkanethiols with minimal damage to the Au nanostructures. Considering the implications of these findings, we are committed to addressing the issue of reusability in our future experiments to assess the feasibility of reusing the developed aptasensor. The incorporation of such investigations in our research endeavors aims to enhance the practicality and versatility of our biosensor technology.

## 4. Conclusions

In summary, we developed and optimized a novel 3D electrode structure for a dual-transducer plasmonic and electrochemical biosensor. The optical transducer operated in transmission mode by utilizing FDTD simulations, and later we compared the simulation results with real experimental data. We found that optical sensing with conventional fully penetrating hole geometries suffers from the overlap of two EOT resonances originating from the Au/substrate and the Au/medium interfaces, which impairs the unambiguous determination of the analyte-associated peak shifts. Hence, nanohole arrays are not ideal for biosensor applications requiring reliable and accurate identification of spectral shifts. In response, we propose eliminating the resonance of the (1,0) Au/substrate mode by introducing nanopit arrays with depressions that do not fully penetrate the metal film and leave a continuous Au film at the Au/substrate interface. The AuNpA not only exhibited a sharper plasmonic spectral width and higher refractive index sensitivity but also enlarged the electrochemical surface area due to their nanotopography. Consequently, in the realization of the plasmonic and electrochemical aptasensor, we found that the electrochemical detection exhibited a wide concentration range from 1 nM to 600 μM, with low sensitivity facilitating HSA screening in a large variety of samples. In contrast, the plasmonic detection ranged from 100 to 600 μM with a high sensitivity, which is ideal for the accurate determination of physiological HSA concentrations. Furthermore, both concentration ranges overlapped and reported on the same binding event in a redundant manner, which enhanced the reliability of the test results. It is noteworthy that these advantages do not come at the cost of a complicated experimental set-up. The electrodes with nanopit arrays were fabricated by an easy-to-perform, large-area nanoimprint lithography process, and the plasmonic sensor used a simple, point-of-care compatible transmission configuration.

To further improve optical sensing in the future, several possibilities can be explored. One method could involve optimizing the design and geometry of the nanopit arrays to achieve even higher sensitivity. Additionally, optimizing the integration of other transduction mechanisms, such as fluorescence or plasmon-enhanced fluorescence (PEF), with the plasmonic sensing platform could open up new avenues for multiplexed and highly sensitive detection. These advancements would contribute to the continuous improvement and broader application of optical sensing technologies. Therefore, we believe that our dual-signal aptasensor with AuNpA possesses great potential in clinical and point-of-care testing applications.

## Figures and Tables

**Table 1:** Sensitivity, transmittance, FWHM and figure of merit of the (1,0) Au/medium mode changing with periodicity.

Periodicity (Lattice Constant) (nm)	Sensitivity (nm/RIU)	Transmittance (%) (RI = 1.0/1.33)	FWHM (nm) (RI = 1.0/1.33)	Figure of Merit (RI = 1.33)
400	205.79	44.2/66.7	253/313	0.66
500	279.36	35.2/72.6	94.4/376	0.74
600	411.15	36.3/32.9	164.9/34.7	11.85
700	587.58	35.4/30.4	278.3/56.0	10.49
800	672.36	33.9/29.0	261.4/73.0	9.21

**Table 2:** Sensitivity, transmittance, FWHM and figure of merit values of the (1,0) Au/medium mode changing with hole depths of 80, 90 and 100 nm.

Hole Depth (nm)	Sensitivity (nm/RIU)	Transmittance (%) (RI = 1.0/1.33)	FWHM (nm) (RI = 1.0/1.33)	Figure of Merit (RI = 1.33)
100	451.82	20.9/22.9	161.0/71.5	6.32
90	474.90	19.4/21.9	113.2/95.1	4.99
80	478.23	11.7/12.8	79.8/71.1	6.73

## Data Availability

The data presented in this study are available upon request from the corresponding author.

## Supplementary Materials

The following supporting information can be downloaded at https://www.mdpi.com/article/10.3390/nano13162374/s1. Figure S1: Fabrication process of the working mold; Figure S2: FDTD simulation spectrum of the fully penetrating AuNhA with different periodicity (Periodicity/Diameter = 1.5) and corresponding refractive index sensitivity of the (1,0) Au/medium mode including 400 nm (a, b), 500 nm (c, d), 600 nm (e, f), 700 nm (g, h), and 800 nm (i, j); Figure S3: SEM cross-section image of the AuNpA by FIB, Title angle = 52°; Figure S4: SEM image of the Si/SiO${}_{2}$ master mold; Figure S5: AFM image of the AuNpA with Cr residue before wet etching; Figure S6: Schematic illustration of the ideal nanopit arrays (a), and experimental ESA comparison of the AuNpA and the compact Au film (b); Figure S7: EIS recorded in 5 mM [Fe(CN)${}_{6}$]${}^{3-/4-}$ Tris buffer at bare AuNpA, AuNpA/Aptamer, AuNpA/Aptamer/6-MCH and adding 1 μM HSA. Solid lines are fitting results obtained with the model of the top right illustration. The electrical equivalent circuit elements used to fit the impedance measurements are shown in the equivalent Randles circuit and the equivalent circuit model used here comprises a resistor for the intrinsic electrolyte resistance (*R*s) in series with a parallel constant phase element (CPE), a charge transfer resistance (*R*ct), and Warburg impedance (W); Figure S8: Cyclic voltammetry (100 continuous cycles with scan rate 100 mV/s) in 5 mM [Fe(CN)${}_{6}$]${}^{3-/4-}$ Tris buffer using the bare AuNpA as working electrode; Table S1: Ormoprime datasheets; Table S2: Dry etching parameters; Table S3: Performance comparison of various strategies for HSA detection; Table S4: Results of the detection of HSA in human serum by standard addition method. References are cited in supplemmentary materils [42,43,44,45,46].
