# Supplementary material for: A Combined Plasmonic and Electrochemical Aptasensor Based on Gold Nanopit Arrays for the Detection of Human Serum Albumin
Source: Nanomaterials (Basel). 2023 Aug 19;13(16):2374. doi: 10.3390/nano13162374 (PMC10458775; doi:10.3390/nano13162374)
Supplement: Supplementary file 1 [file nanomaterials-13-02374-s001.zip › nanomaterials-2534926-supplementary.pdf]

A Combined Plasmonic and Electrochemical Aptasensor Based on Gold Nanopit Arrays for the Detection of Human Serum Albumin

Ruifeng Zhu[1,2], Gabriela Figueroa-Miranda[1], Lei Zhou[1], Ziheng Hu[1], Bohdan Lenyk[1,3], Sven Ingebrandt[2], Andreas Offenhäusser[1], and Dirk Mayer *[1]

[1] *Institute of Biological Information Processing, Bioelectronics (IBI-3), Forschungszentrum Jülich GmbH, 52428 Jülich, Germany*

[2]*Institute of Materials in Electrical Engineering 1, RWTH Aachen University, 52074 Aachen, Germany*

[3]*Department of Physics, University of Konstanz, 78457 Konstanz, Germany*

*Corresponding author, email: dirk.mayer@fz-juelich.de

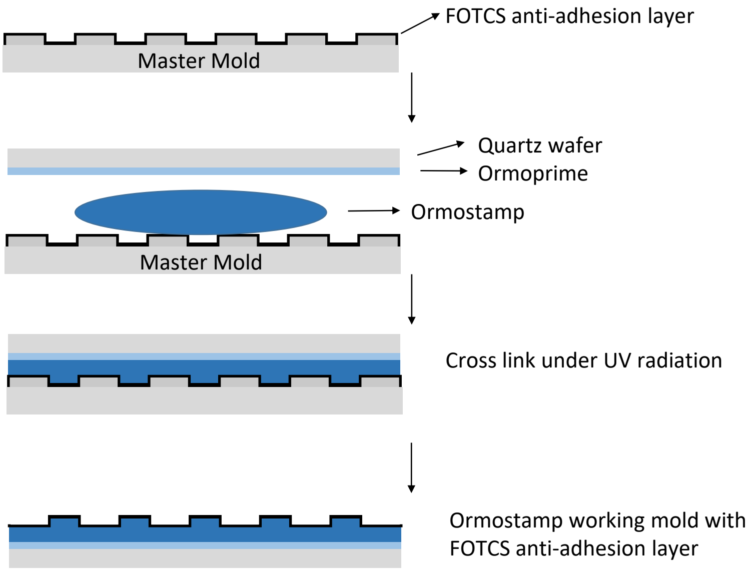

Figure S1: Fabrication process of the working mold.

Table S1: Ormoprime datasheets.

Ormoprime	
Spin coating	
spin speed [rpm]:	4000
time [s]:	60
acceleration [rpm/s]:	1000
hardbake [°C]:	150
time [min]:	5
film thickness [nm]:	130 ± 15

Table S2: Dry etching parameters.

Cr etch recipe		Au etch recipe	
Cl_2 gas flow	50 sccm	Cl_2 gas flow	15 sccm
O_2 gas flow	3 sccm	Ar gas flow	5 sccm
RF power	150 W	RF power	50 W
ICP power	0 W	ICP power	800 W
Chamber pressure	0.09 Torr	Chamber pressure	0.007 Torr
Temperature	30 °C	Temperature	50 °C
Etching rate for mr-NIL200	176 nm/min	Etching rate for Cr	13 nm/min
Etching rate for Cr	39 nm/min	Etching rate for Au	72 nm/min
Selectivity (mr-NIL200/Cr)	4.51	Selectivity (Cr/Au)	0.18

Table S3: Performance comparison of various strategies for HSA detection.

Biosensor structure or surface modification	Method	Dynamic range of detection	Limit of detection	Reference
Single-walled carbon nanotube with antibodies	FET biosensors	1 fg/mL - 10 pg/mL	0.47 fg/mL	[1]
Gold nanoclusters capped glutathione as a fluorescent probe	Fluorescence	6 μM - 338 μM	4 μM	[2]
CuInZnS quantum dots-Co^{2+} sensing system	Photoluminescence	75 nM - 100 μM	48 nM	[3]
Cabergoline-HSA interactions	Amperometric method	1 - 40 μM	0.6 μM	[4]
Curcumin embedded in bacterial cellulose nanopaper	Naked eye / Smartphone camera by optical sensing	10 - 300 μM / 25 - 400 μM	5 μM / 5 μM	[5]
Gold nanopit arrays with HSA aptamer	Plasmonic / Electrochemical aptasensor	100 - 600 μM / 0.1 nM - 600 μM	95.22 μM / 0.08 nM	This work

Table S4: Results of the detection of HSA in human serum by standard addition method.

1% Human Serum	Spiked	Measured		Recovery		RSD	
Sample	uM	Plasmonic	Electrochemical	Plasmonic	Electrochemical	Plasmonic	Electrochemical
1	0	-	5.54	-	-	-	12.54%
2	100	100.58	87.67	95.30%	83.07%	12.76%	4.12%
3	200	177.15	163.95	86.19%	79.77%	28.38%	5.68%

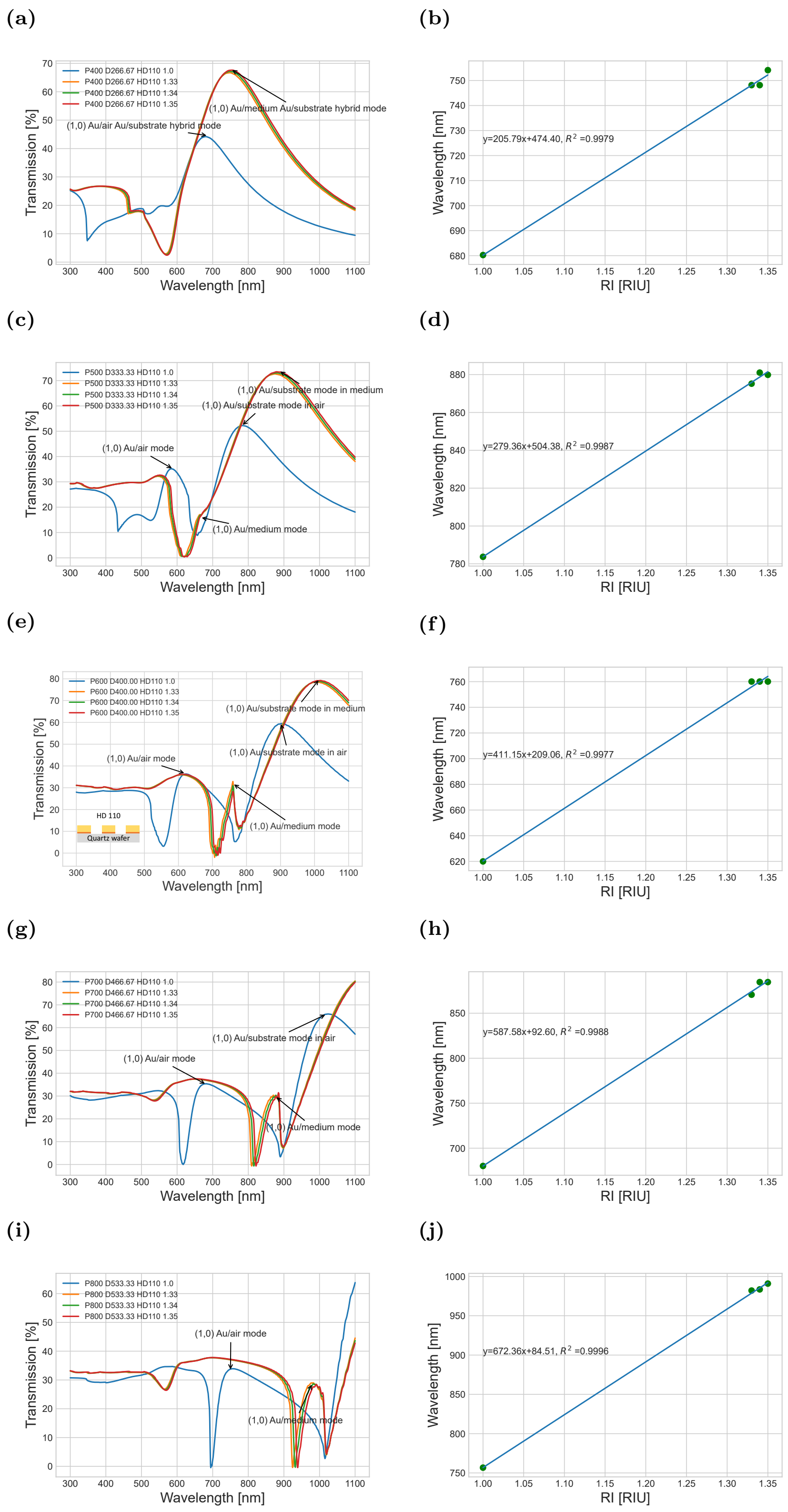

Figure S2: FDTD simulation spectrum of the fully penetrating AuNhA with different periodicity (Periodicity/Diameter = 1.5) and corresponding refractive index sensitivity of the (1,0) Au/medium mode including 400 nm (a, b), 500 nm (c, d), 600 nm (e, f), 700 nm (g, h), and 800 nm (i, j).

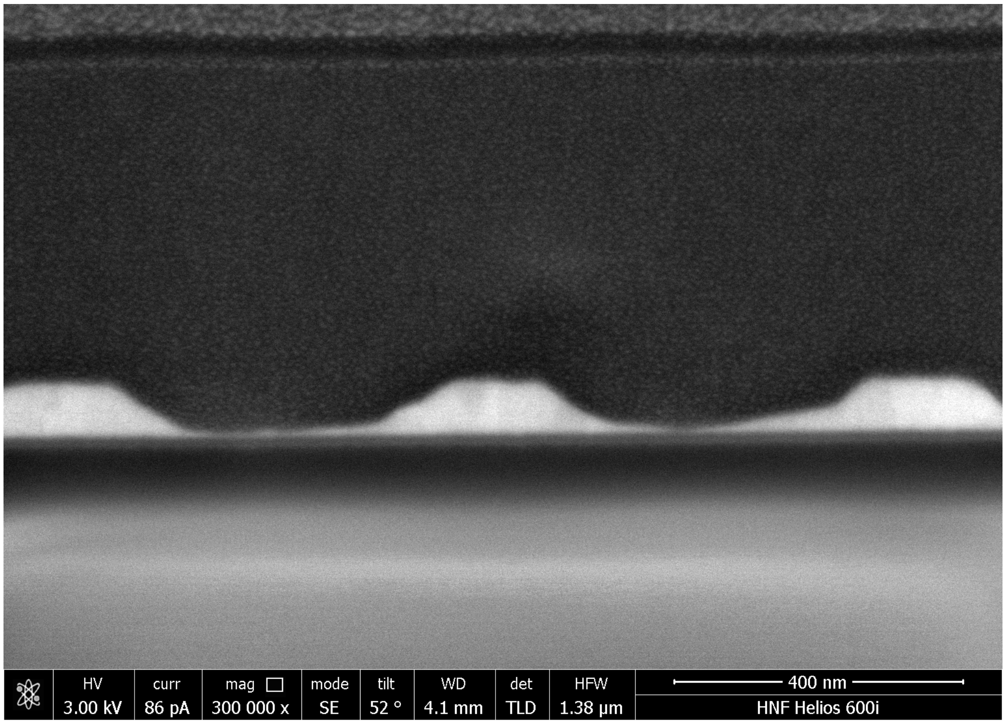

Figure S3: SEM cross-section image of the AuNpA by FIB, Title angle = 52°.

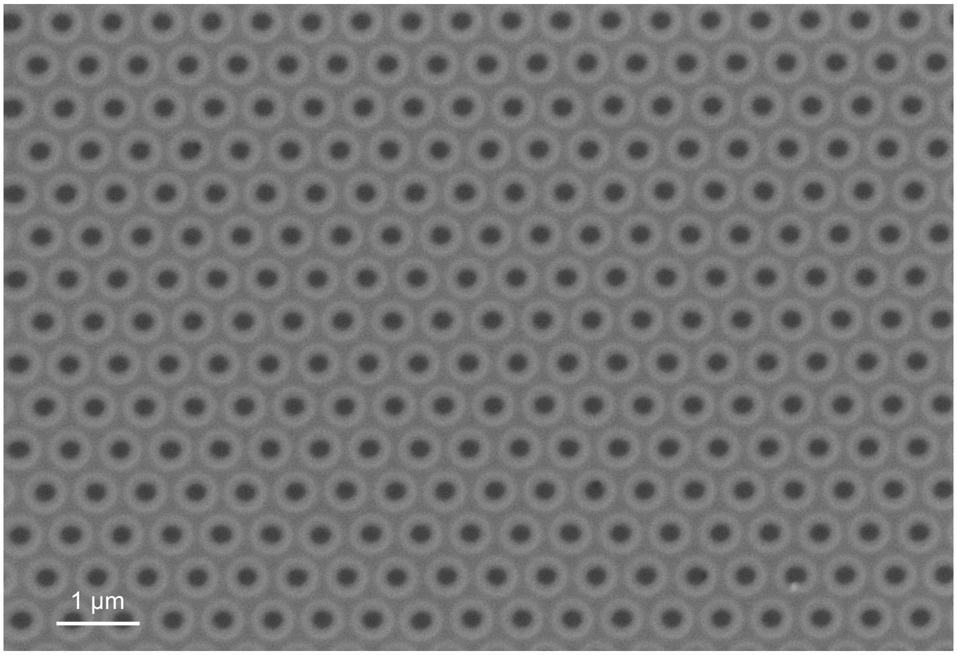

Figure S4: SEM image of the Si/SiO_2 master mold.

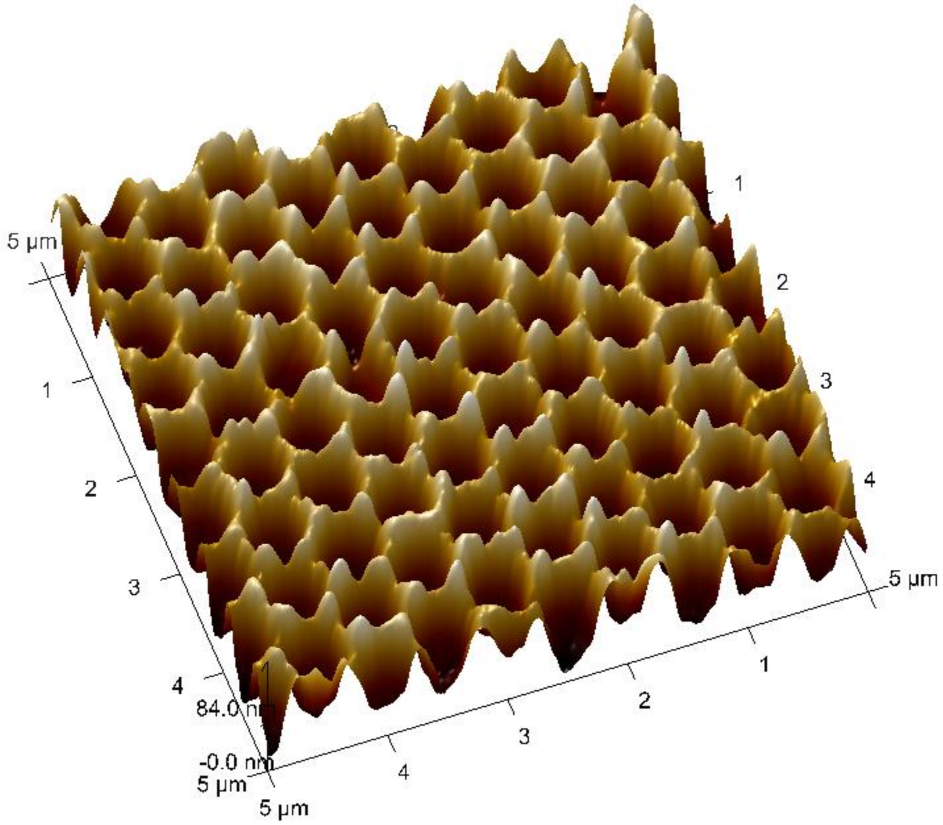

Figure S5: AFM image of the AuNpA with Cr residue before wet etching.

(a)

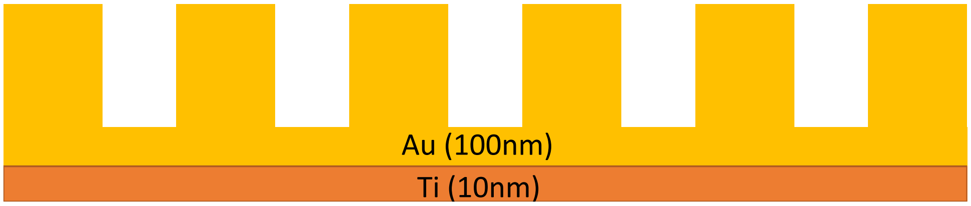

(b)

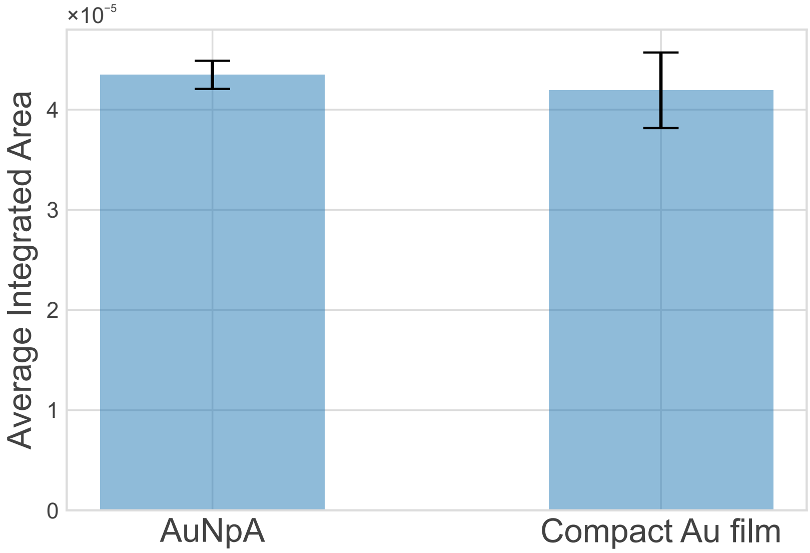

Figure S6: Schematic illustration of the ideal nanopit arrays (a), and experimental ESA comparison of the AuNpA and the compact Au film (b).

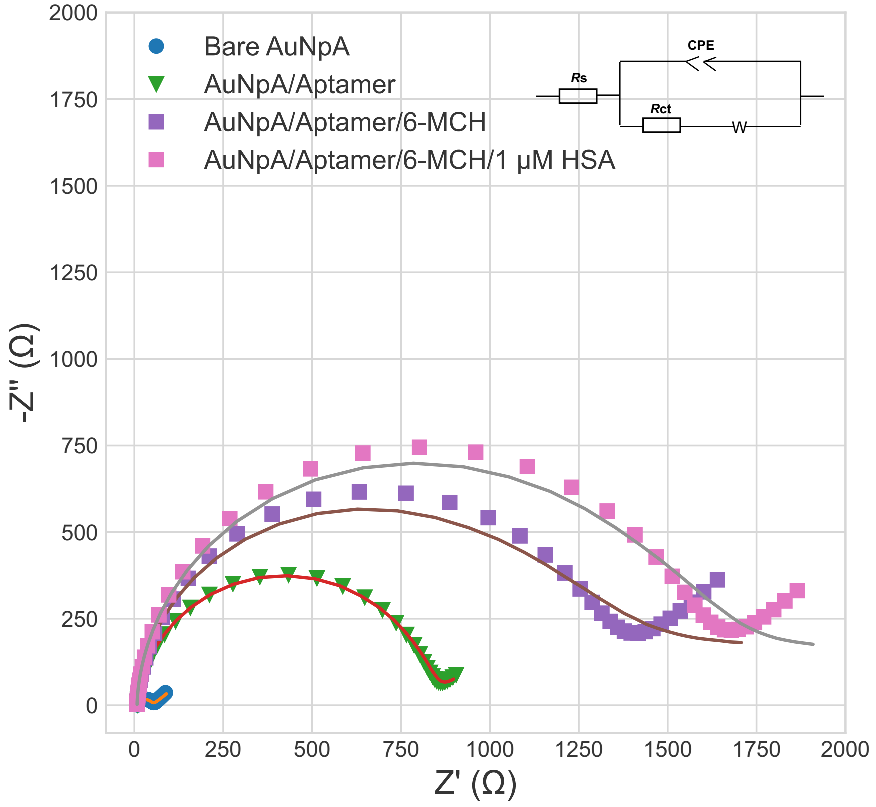

Figure S7: EIS recorded in 5 mM $[Fe(CN)_6]^{3-/4-}$ Tris buffer at bare AuNpA, AuNpA/Aptamer, AuNpA/Aptamer/6-MCH and adding 1 μM HSA. Solid lines are fitting results obtained with the model of the top right illustration. The electrical equivalent circuit elements used to fit the impedance measurements are shown in the equivalent Randles circuit and the equivalent circuit model used here comprises a resistor for the intrinsic electrolyte resistance (Rs) in series with a parallel constant phase element (CPE), a charge transfer resistance (Rct), and Warburg impedance (W).

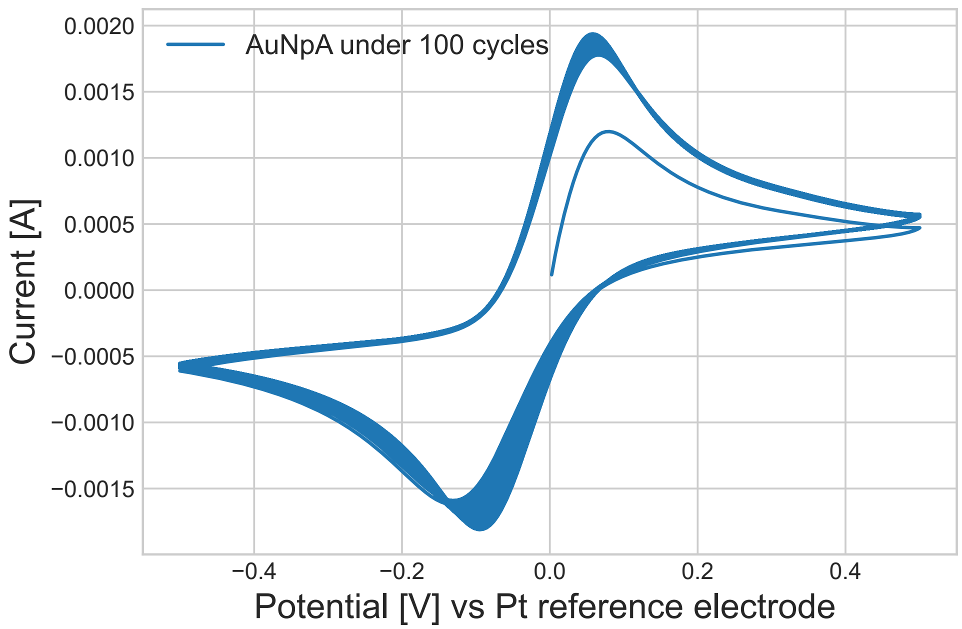

Figure S8: Cyclic voltammetry (100 continuous cycles with scan rate 100 mV/s) in 5 mM $[Fe(CN)_6]^{3-/4-}$ Tris buffer using the bare AuNpA as working electrode.

References

[1] Iskandar Yahya, Muhamad Azuddin Hassan, Nur Nasyifa Mohd Maidin, and Mohd Ambri Mohamed. Swcnt network-fet device for human serum albumin detection. *Sensors*, 22(21), 2022.

[2] Wenxiang Xiao, Yaoxin Li, Yinan Xiong, ZhenCheng Chen, and Hua Li. Fluorescence turn-on detection of human serum albumin based on the assembly of gold nanoclusters and bromocresol green. *Analytical and Bioanalytical Chemistry*, 415(17):3363–3374, 2023.

[3] Wenying Gui, Xueqian Chen, and Qiang Ma. A novel detection method of human serum albumin based on cuinzns quantum dots-co2+ sensing system. *Analytical and Bioanalytical Chemistry*, 409(15):3871–3876, 2017.

[4] Ali R. Jalalvand, Sirous Ghobadi, Vali Akbari, Hector C. Goicoechea, Elahe Faramarzi, and Majid Mahmoudi. Mathematical modeling of interactions of cabergoline with human serum albumin for biosensing of human serum albumin. *Sensing and Bio-Sensing Research*, 25:100297, 2019.

[5] Tina Naghdi, Hamed Golmohammadi, Maryam Vosough, Mojgan Atashi, Iman Saeedi, and Mohammad Taghi Maghsoudi. Lab-on-nanopaper: An optical sensing bioplatform based on curcumin embedded in bacterial nanocellulose as an albumin assay kit. *Analytica Chimica Acta*, 1070:104–111, 2019.
